# Associating the Change in New COVID-19 Cases to GDP per Capita in 38 European Countries in the First Wave of the Pandemic

**DOI:** 10.3389/fpubh.2020.582140

**Published:** 2021-01-20

**Authors:** Shahina Pardhan, Nick Drydakis

**Affiliations:** ^1^Vision and Eye Research Institute, School of Medicine, Anglia Ruskin University, Cambridge, United Kingdom; ^2^Centre for Pluralist Economics, Faculty of Business and Law, Anglia Ruskin University, Cambridge, United Kingdom; ^3^Pembroke College, University of Cambridge, Cambridge, United Kingdom; ^4^Institute of Labor Economics, Bonn, Germany; ^5^Global Labor Organization, Essen, Germany

**Keywords:** COVID-19, GDP per capita, life expectancy, sanitation, smoking

## Abstract

COVID-19 has affected all countries globally. We explore associations between the change in new COVID-19 registered cases per million population and various macroeconomic and well-being indicators in 38 European countries over a 2-month period (1st April-31st May 2020). A statistically significant (*p* = 0.002) negative association was estimated between the change in new COVID-19 cases and GDP per capita, after controlling for key health determinants including public expenditure on health, life expectancy, smoking tobacco and sanitation. The country with the highest GDP per capita in Europe (i.e., Luxemburg) was found to experience the lowest change in new COVID-19 cases within the time period whilst the opposite was found for countries with lower GDP per capita (i.e., Ukraine, Bulgaria, and Romania). The outcomes of this study indicate that, in the first wave of the pandemic in Europe, a country's GDP per capita might be associated with a lower rate of new COVID-19 cases. The study concludes by suggesting that in European regions a country's economic performance should be a critical health priority for policy makers.

## Introduction

The global pandemic caused by COVID-19 has affected every county in the world (1). Various factors have been shown to influence the rate of change of infection caused by SARS CoV-2-s as confirmed by the daily change in new cases and of mortality. Individual and demographic factors including older age (2, 3), male gender (4), socio-economic conditions (5), underlying health co-morbidities (6), ethnicity (7, 8), smoking (9, 10) and obesity (11) have been identified as significant influencing factors in the spread and mortality due to COVID-19. However, it has to be recognized that these individual factors are likely to be influenced by various country specific parameters including lockdown policies (12), public expenditure in health (13) and other country-specific determinants such as level of sanitation (14), healthcare support i.e., number of acute care beds, hospital beds and number of physicians; (15), and social support (16). Grima et al. (17) indicated that monitoring of demographic features, country's activity features, economic exposure and societal vulnerability could help a country strengthen its capacity to meet the economic, social and in turn healthcare demands due to pandemic hazards such as COVID-19.

In poorer countries, households experience tighter budgets and worse economic struggles (18, 19), resulting in poor physical and mental health and decreased life expectancy (18, 20). One of the most well-known illustrations of the relationship between economic conditions and population health is the Preston curve (21) which demonstrates that people in richer countries, on average, live longer than in poorer countries. Evidence indicates that a country's economic performance and fair income distribution results in an increase in life expectancy, and a decrease in adverse health outcomes and mortality rates (22). Theoretical insights indicate that countries with higher GDP rates are able to provide their people with better living standards, public health programs, education and environmental sanitation all leading to enhanced prevention, treatment of disease, better health and life expectancy (17, 22–26).

In the US, GDP and economic growth were found to be related to a decline in mortality rate between 1901 and 2000 (27). In OECD countries, for a period ranging from 1820 to 2001, GDP and GDP per capita were estimated to have a significant positive influence on life expectancy (28). In Europe, longitudinal studies, review studies and meta-analyses indicate that reductions in GDP, national health system budgets and households' income are associated with adverse health outcomes and deterioration in people's well-being (29–31). The Great Recession studies re-highlighted the positive associations of economic prosperity and adequate national health provision to better health outcomes (29–31). In Europe, since 1950, the rapid economic growth brought a degree of prosperity which enabled effective national health systems to be established, new drugs and medical technologies to be developed that brought several infections and diseases under control (32). However, better health systems, cutting-edge medical technologies and drugs required substantial funding (32). Within the World Health Organization (WHO) European regions, for a period ranging from 1900 to 2008, life expectancy was shown to be dependent on economic activity, and mortality from cardiovascular diseases were mainly attributable to changes in national income (23). These patterns have been found in other diverse settings. For instance, a recent study of young population groups in 103 low and middle-income countries showed that higher GDP was inversely associated with all-cause, communicable and non-communicable disease mortality both in males and females (33). A study examining empirical assessments for 17 European countries between 1970 and 2010, demonstrated that countries with higher national income, higher health care expenditure, higher quality of government, and higher social transfers have smaller inequalities in mortality (34).

Given the presented theoretical considerations and empirical findings, we hypothesize that poorer countries in Europe, characterized by a lower economic performance, and limited health and fiscal capacities of governments might underperform in tackling the COVID-19 pandemic. Restricted personal and family income, and inadequate public support for critical health services could affect health prevention and the quality and quantity of services experienced during the pandemic. In the US, those affected more by COVID-19 were living in poorer regions, had lower access to healthcare, experienced intergenerational poverty, and had a higher prevalence of underlying health conditions (35, 36). A recent study utilizing data from 188 countries found that COVID-19 has mainly affected vulnerable population groups with underlying health conditions (37).

Although GDP per capita is one of the most widely used covariates in health research (38), most current papers on COVID-19 have focused on individuals' socio-economic and health characteristics (35–37). However, there are few exceptions. Roy (39) presented a diagrammatic reasoning demonstrating a negative relationship between the total number of COVID-19 cases and GDP per capita. On the other hand, Gangemi et al. (40) found a moderate positive correlation between COVID-19 cases and GDP per capita as did Lippi et al. (41) who showed a positive correlation between COVID-19 mortality and GDP per capita. The studies suggested that industrial pollution, airplane connections, obesity and social events, which are higher in developed regions, might have driven the positive correlations (40, 41).

The aim of the present study is to explore correlations and associations between the change in new COVID-19 cases per million population, in the initial wave of the pandemic, obtained on two dates that were 2 months apart (1st April and 31st May 2020), and macroeconomic and well-being indicators in 38 European countries. In this study we indicate that the total number of COVID-19 cases may not be the right indicator to be utilized because case zero of COVID-19 case differs in each country as the pandemic affected regions at different times. In addition, we hypothesize that the change in new COVID-19 cases per million population might be more detrimental in regions where prior to the pandemic, people were experiencing worse health due to greater economic hardships and insecurity.

The present paper is among the first studies to offer multivariate regressions controlling for key heterogeneities and assess associations between GDP per capita and change in new COVID-19 cases in European regions in the first wave of the pandemic. The outcomes of the study will show (i) a negative correlation between the change in new COVID-19 cases per million population and GDP per capita, public expenditure on health, sanitation facilities and life expectancy at birth, and (ii) a positive correlation between the change in new COVID-19 cases and tobacco smoking. Moreover, the study's outcomes will reveal a negative association between the change in new COVID-19 cases per million population and GDP per capita after controlling for critical health heterogeneities. The study will conclude that in Europe a country's prosperity, as it is captured by GDP per capita, might be associated with a reduction in new COVID-19 cases within the study's time period. The assigned patterns will indicate that better economically performed countries might be able to respond to a health crisis and therefore a country's economic growth and development should be of importance. Indeed, the study will present that the best performing European country in terms of GDP per capita (i.e., Luxemburg) experienced the lowest change in new COVID-19 cases per million population.

The rest of the paper is structured as follows. In the next section we describe the data set and methodology. Then, we offer a correlation and regression analysis. The last section offers a discussion and conclusions.

## Materials and Methods

In this study data on the change in new COVID-19 cases per million population were obtained for 38 European countries, as some of the European data required for correlation and multivariate analyses were only available for certain countries^1^. In June 2020, COVID-19 data were extracted from Roser et al. (42) which is a publicly available data set.

Key macroeconomic and well-being indicators for the targeted European countries were considered after reviewing major socio-epidemiological surveys (14, 22, 29, 37). These variables constitute critical health determinants such as GDP per capita, smoking tobacco, sanitation facilities, alcohol consumption, acute care and total number of hospital beds (22). The variables were extracted from major publicly available data sets^2^. For each parameter, the last recorded year's entry was used for each country (if relevant information was available). As the study utilized publicly available data, ethical clearance was not required.

We indicate that while previous studies have compared the total number of COVID-19 cases per million population registered on a certain date, this approach might be inaccurate as different European countries reported their first COVID-19 case at different times and therefore some European countries would probably show higher prevalence compared to others. In the present study we calculate the change in the numbers of new COVID-19 cases between two dates, which were 2 months apart, i.e., 1st April and 31st May 2020. As an example, the change of new COVID-19 cases in the UK within the study's time period is −1,405 COVID-19 cases (42).

## Results

The empirical specification of this study consists of two parts. In the first part, we present a correlation analysis, also used by Gangemi et al. (40) and Lippi et al. (41), between the change in new COVID-19 cases per million population and macroeconomic and well-being indicators. Given the nature of the variables (continuous) Pearson correlation coefficients are reported. In the second part, we offer an OLS regression analysis assessing the determinants of change in new COVID-19 cases per million population. The regressions control for critical determinants of COVID-19 disease (35–37). These are GDP per capita, public expenditure on health, life expectancy at birth, smoking tobacco, and sanitation facilities. In the regression analysis, the main interest is to assess whether GDP per capita is associated with the change in new Covid-19 cases per million population. If GDP per capita remains statistically significant after controlling for key covariates, then this feature might indicate an association between the change in new COVID-19 cases per million population and countries' economic performance in the specified region and period.

In this study we highlight that the regression outcomes should be interpreted as associations and not as causal effects. It is well-documented in the literature that a two-way relationship between a country's performance and good health might exist (22) due to the fact that health may actually drive economic performance (28, 31).

### Correlation and Regression Outcomes

#### Correlation Analysis

Table 1 shows correlation coefficients for the change in new COVID-19 cases per million population. Statistically significant negative correlations were obtained between the change in new COVID-19 cases and GDP per capita, public expenditure on health, sanitation facilities and life expectancy at birth. The outcomes indicate a country's higher prosperity (GDP per capita), public spending on health, and well-being indicators (such as sanitation infrastructures and life expectancy at birth) is associated with a lower change in new COVID-19 per million population. A statistically significant positive correlation between the change in new COVID-19 cases and tobacco smoking is also found, indicating that smoking prevalence might deteriorate health status and/or be an underlying health co-morbidity.

**Table 1:** Correlation coefficients between the change in new COVID-19 cases per million population and socio-epidemiological determinants over a two-month period (1st April 2020 and 31st May 2020).

	**Panel I** **Change in new** **COVID-19 cases** **per million population**	**Number of** **countries**
GDP per capita	−0.665(0.001)^***^	37^a^
Extreme poverty	0.135(0.503)	27^b^
Public expenditure on health	−0.557(0.001)^***^	38
Obesity	0.077(0.644)	38
Overweight	−0.194(0.314)	29^c^
Social support	−0.252(0.126)	38
Smoking tobacco	0.344(0.035)^**^	38
Alcohol	−0.127(0.453)	37^d^
Life expectancy at birth	−0.565(0.001)^***^	38
Sanitation facilities	−0.409(0.012)^**^	37^e^
Acute care beds	0.210(0.212)	37^f^
No of Hospital beds	0.231(0.162)	38
Median Age	0.032(0.849)	37^g^
No physicians	−0.058(0.731)	37^h^

*Pearson correlation coefficients are reported. p-values are in parentheses*.

a *Andorra is excluded due to data unavailability*.

b *Andorra, Cyprus, the Czech Republic, Finland, France, Germany, the Netherlands, Poland, Serbia, Slovenia, and Switzerland are excluded due to data unavailability*.

c *Bulgaria, Croatia, Germany, Lithuania, Luxemburg, Spain, Sweden, Switzerland and the United Kingdom are excluded due to data unavailability*.

d *Germany is excluded due to data unavailability*.

e *Montenegro is excluded due to data unavailability*.

f *Bosnia and Herzegovina is excluded due to data unavailability*.

g *Andorra is excluded due to data unavailability*.

h *Slovakia is excluded due to data unavailability*.

*** *Statistically significant at the 1%*.

** *Statistically significant at the 5%*.

**Figure 1:**
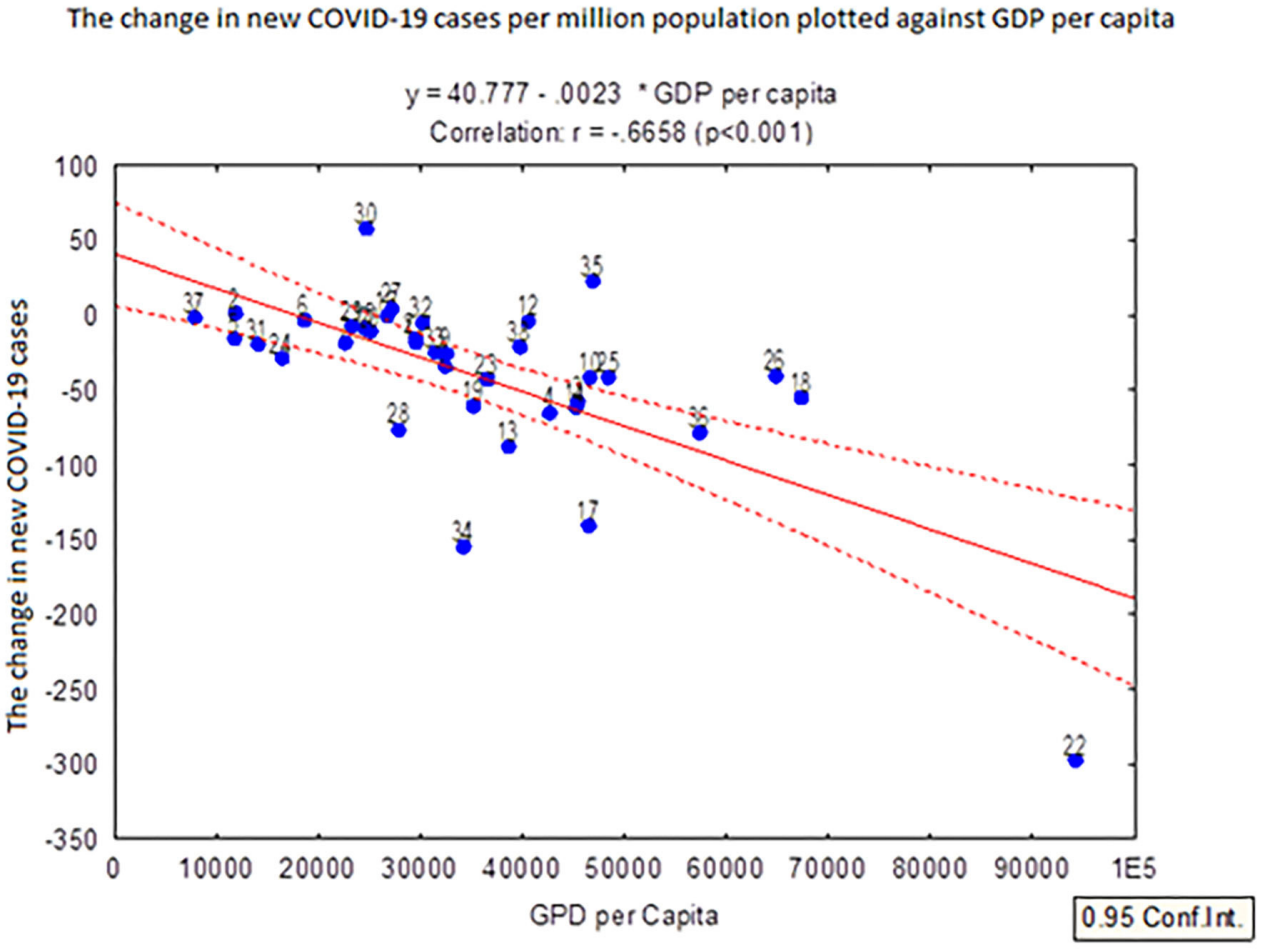
The change in new COVID-19 cases per million population over a 2-month period (1st April and 31st May 2020) plotted against the GDP per capita in 38 European countries. The countries denoted in the figure are: 1: Albania; 2: Andorra; 3: Austria; 4: Belgium; 5: Bosnia and Herzegovina; 6: Bulgaria; 7: Croatia; 8: Cyprus; 9: the Czech Republic; 10: Denmark; 11: Estonia; 12: Finland; 13: France; 14: Germany; 15: Greece; 16: Hungary; 17: Iceland; 18: Ireland; 19: Italy; 20: Latvia; 21: Lithuania; 22: Luxemburg; 23: Malta; 24: Montenegro; 25: the Netherlands: 26: Norway; 27: Poland; 28: Portugal; 29: Romania; 30: Russia; 31: Serbia; 32: Slovakia; 33: Slovenia; 34: Spain; 35: Sweden; 36: Switzerland; 37: Ukraine; and 38: the United Kingdom. For example, point 22 is Luxembourg.

Figure 1 presents a negative correlation between the change in new COVID-19 cases per million population and GDP per capita. The figure indicates that Luxemburg which was the best performing European country in terms of GDP per capita also experienced the lowest change in new COVID-19 cases per million population. On the other hand, countries facing lower GDP per capita, such as Ukraine, Bulgaria, Romania and Russia, experienced a higher level of change in new COVID-19 cases per million population. The country with the highest level of change in new COVID-19 cases per million population, within the time period, was Russia.

#### Regression Outcomes

Table 2 presents the regression outcomes. We include only those variables that were found to be statistically significant with the change in new COVID-19 cases in Table 1 (i.e., GDP per capita, public expenditure on health, life expectancy at birth, smoking tobacco and sanitation facilities).

**Table 2:** Regression outcomes: The change in new COVID-19 cases per million population.

	**Panel I** **Change in new** **COVID-19 cases** **per million** **population**	**Panel II** **Change in new** **COVID-19 cases** **per million** **population**	**Panel III** **Change in new** **COVID-19 cases** **per million** **population**
GDP per capita	−0.810 (0.005)^***^	−0.801 (0.004)^***^	−0.878 (0.002)^***^
Public expenditure on health	0.164 (0.540)	0.308 (0.296)	0.365 (0.215)
Life expectancy at birth	–	−0.346 (0.060)	−0.188 (0.383)
Smoking tobacco	–	−0.141 (0.390)	−0.120 (0.467)
Sanitation facilities	–	–	−0.218 (0.212)
Adjusted *R*^2^	0.417	0.440	0.462
*F*	13.86	8.312	7.017
*p*	0.000	0.001	0.001

*N = 36. Andorra and Bosnia and Herzegovina are excluded due to data unavailability. p-values are in parentheses*.

*** *Statistically significant at the 1%*.

Panel I controls for public expenditure on health. The estimates indicate that GDP per capita is negatively associated with the change in new COVID-19 cases per million population at the 1% level. In Panel II, we control also for life expectancy at birth and smoking tobacco. The new estimates continue to indicate a negative association between GDP per capita and the change in new COVID-19 cases per million population at the 1%. In Panel III, we add controls for sanitation facilities. It is found that GDP per capita continues to be negatively associated with the change in new COVID-19 cases per million population at the 1%.

## Discussion

The aim of the study was to examine correlations between the change in new COVID-19 cases per million population and macroeconomic indicators, well-being indicators and healthcare systems' capacity in the first wave of the COVID-19 pandemic. Most recent studies have focused on demographic, socio-economic and health characteristics and the COVID-19 pandemic (2, 6, 7, 17). In this study we were particularly interested in assessing whether countries' economic performance, as it is captured by GDP per capita, might be associated with COVID-19 spread in 38 European countries, in the early stages. By extracting data in June 2020 and calculating the change in new COVID-19 cases over a 2-month period, between 1st April and 31st May 2020, univariate correlation analysis indicated that new COVID-19 cases per million population were (i) negatively correlated with GDP per capita, public expenditure on health, sanitation facilities and life expectancy at birth and (ii) positively correlated with tobacco smoking. The adjusted regression analysis shows a significant negative association between the change in new COVID-19 cases per million population and GDP per capita. The outcomes of this study are in line with the limited research indicating that COVID-19 cases are negatively associated with public expenditure in health and health care infrastructure and capacity (13, 15), sanitation (14) and positively associated with smoking habits (9, 10).

The study contributes to the literature by assessing both correlations and associations between COVID-19 and GDP per capita. While recent studies have examined associations between COVID-19 and individuals' income (35–37), there is little information assessing a country's economic performance with COVID-19 cases (39). Our study shows a negative relationship between the change in new COVID-19 cases and GDP per capita, in the first wave of the pandemic. We indicate that GDP per capita differences among European countries might reflect existing structural and economic factors (18). People living in European countries with lower GDP per capita might have poorer access to health services and have lower income resulting in poorer health (31). These factors might be exaggerated during periods of severe health and economic crises, negatively affecting the less developed regions (31).

Prior to the pandemic, socio-epidemiological research indicated positive associations between a country's economic performance and better health (22, 27, 28, 34), as well as positive associations between individuals' income and health status (18, 20). In the present study we indicate that a wealthier country might be able to provide their people with better living standards, public health and environmental sanitation, leading to enhanced prevention and disease treatment (22–24).

The last fifty years, European countries have experienced a massive economic growth that have enabled them to invest in health and develop effective health systems and brought several infections and diseases under control (22, 24, 32). We should note that a country's economic performance not only determines health systems' capacity and effectiveness but also its labor market and vocational relations within the country. It is possible that in advanced European countries, lockdowns to save lives might be easier since a greater number of people work in sectors where information technology infrastructure enables them to work from home (43). This might be challenging in poorer European countries where a greater number of people work in sectors where manual labor is needed (43). Such jobs make up the new COVID-19 essential workforce i.e., food service workers, bus drivers.

There are a number of limitations in the present study. It is possible that a change in the number of new COVID-19 cases may not be an accurate reflection of the true infection rate, as testing of infection may be different in various countries. An unbiased estimate of the infection rate will be only possible at the end of the pandemic and/or when there are no deaths reported due to COVID-19. As the data set captured a certain time interval, the choice of the specified time-period did not take into account any lockdown effects in each country or the phase of infection in that country. In addition, the pandemic has carried on so analysis may be different for different time frames. Future studies should utilize different time series data in order to provide longitudinal evaluations.

A further limitation of the present study is that our data were from European countries. New research should consider other parts of the world for firm evaluations. In addition, in the present study, only limited number of available macroeconomic variables were analyzed. It would of interest to examine whether a country's wealth, income distribution, and saving per capita might be associated with COVID-19 spread. We should emphasize that a country's performance is not the only parameter that influences the spread of COVID-19. Other health and social determinants should be considered, as well. In the US, Millett et al. (36) found disproportionately higher rates of COVID-cases and deaths in black counties compared with other counties. This is also shown in the UK with higher prevalence of COVID-19 in Black, Asian and Minority ethnicities (44). People living in deprived areas also experienced worse health (45, 46). Hence, it would be of importance for new research to consider interactions between economic indicators at a microeconomic and macroeconomic level and ethnicity, gender, underlying health conditions, labor characteristics and mobility during the lockdown and their associations with COVID-19 spread.

Moreover, new studies might examine possible interactions between GDP per capita and the level of industrial pollution and urban segregation, and their associations with COVID-19 cases. For instance, in Italy, mortality due to COVID-19 was found to be higher in areas with higher GDP per capita (41). The study suggested that adverse environmental factor such as higher industrial pollution, in addition to the other known risk factors such as obesity and hypertension, might have driven the outcomes (41).

It is known that many other factors influenced the rate of COVID-19 infections. In Wuhan-China, a positive impact of lockdown was found to restrain further increases of COVID-19 cases (12). In addition to the social, health and economic determinants, it is possible that a “level of preparedness” may also play a significant role in reducing COVID-19 spread. Research suggests that COVID-19 prevalence and mortality rates were lower in African and Asian countries compared to certain Western European countries and the US (47). Several factors may have contributed to this including early instigation of lockdown and border closures, younger age of the population, lack of robust reporting systems, and other unidentified genetic factors (47). It is possible that some countries might be better prepared to deal with COVID-19 spread because they have accumulated experiences from previous pandemics including SARS. For example, Ghana initiated lockdowns within weeks of the first COVID-19 cases and was ranked number one among African countries in administering tests per million people (48). Fan et al. (49) reporting raw case fatality rate of 53 countries with the highest COVID-19 death tolls, showed that 43 countries had lower raw case fatality rate estimates in the second wave of the COVID-19 pandemic, indicating that healthcare system of the countries might have been better prepared for the second phase (49, 50). The theory of better immunity in economically poor countries was considered by Roy (39) who showed that, in a sample of 46 countries, although the number of new COVID-19 cases showed a slight decline in lower-income countries, the fatality rate was independent of the financial condition of the countries in question.

Our study shows that GDP per capita might be a critical epidemiological parameter when comparing different countries within Europe. We reported a statistically significant negative association between GDP per capita and the change in new cases of COVID-19 per million population during the first wave over a 2-month period (1st April-31st May 2020) in 38 European countries. The outcomes of this study should call the attention of policy makers. If better economically performed economies in Europe display lower susceptibility to pandemics and to new infections in the early stages of the pandemic, a country's economic growth and development should be perceived as a vital priority for policy makers in future pandemics.

## Data Availability Statement

Publicly available datasets were analyzed in this study. This data can be found here: https://ourworldindata.org/coronavirus; Public expenditure on health: http://dmt.euro.who.int/classifications/tree/A; Prevalence of obesity: https://gateway.euro.who.int/en/indicators/h2020_9-obesity/; Prevalence of overweight: https://gateway.euro.who.int/en/indicators/h2020_6-overweight/; Availability of social support: http://dmt.euro.who.int/classifications/tree/A; Smoking prevalence: http://dmt.euro.who.int/classifications/tree/A; Alcohol consumption: http://dmt.euro.who.int/classifications/tree/B; Life expectancy at birth: http://dmt.euro.who.int/classifications/tree/B; Sanitation facilities: http://dmt.euro.who.int/classifications/tree/B; Acute care and total number of hospital beds: http://dmt.euro.who.int/classifications/tree/A; Practicing physicians: http://dmt.euro.who.int/classifications/tree/L.

## Ethics Statement

Ethical review and approval was not required for the study on human participants in accordance with the local legislation and institutional requirements. Written informed consent for participation was not required for this study in accordance with the national legislation and the institutional requirements.

## Author Contributions

Both authors contributed to the whole paper. SP carried out data analysis. SP and ND contributed equally to introduction, results, and discussion.

## Conflict of Interest

The authors declare that the research was conducted in the absence of any commercial or financial relationships that could be construed as a potential conflict of interest.

## Footnotes

^1^1: Albania; 2: Andorra; 3: Austria 4: Belgium 5: Bosnia and Herzegovina; 6: Bulgaria; 7: Croatia; 8: Cyprus; 9: the Czech Republic; 10: Denmark; 11: Estonia; 12: Finland; 13: France; 14: Germany; 15: Greece; 16: Hungary; 17: Iceland; 18: Ireland; 19: Italy; 20: Latvia; 21: Lithuania; 22: Luxemburg; 23: Malta; 24: Montenegro; 25: the Netherlands: 26: Norway; 27: Poland; 28: Portugal; 29: Romania; 30: Russia; 31: Serbia; 32: Slovakia; 33: Slovenia; 34: Spain; 35: Sweden; 36: Switzerland; 37: Ukraine; 38: the United Kingdom.

^2^Gross Domestic Product per capita and extreme poverty: https://ourworldindata.org/coronavirus; Public expenditure on health: http://dmt.euro.who.int/classifications/tree/A; Prevalence of obesity: https://gateway.euro.who.int/en/indicators/h2020_9-obesity/; Prevalence of overweight: https://gateway.euro.who.int/en/indicators/h2020_6-overweight/; Availability of social support: http://dmt.euro.who.int/classifications/tree/A; Smoking prevalence: http://dmt.euro.who.int/classifications/tree/A; Alcohol consumption: http://dmt.euro.who.int/classifications/tree/B; Life expectancy at birth: http://dmt.euro.who.int/classifications/tree/B; Sanitation facilities: http://dmt.euro.who.int/classifications/tree/B; Acute care and total number of hospital beds: http://dmt.euro.who.int/classifications/tree/A; Practicing physicians: http://dmt.euro.who.int/classifications/tree/L.
